# Global analysis of host response to induction of a latent bacteriophage

**DOI:** 10.1186/1471-2180-7-82

**Published:** 2007-08-31

**Authors:** Robin E Osterhout, Israel A Figueroa, Jay D Keasling, Adam P Arkin

**Affiliations:** 1 Department of Chemistry, University of California at Berkeley, Berkeley, CA 94720, USA; 2 Department of Bioengineering and Howard Hughes Medical Institute, University of California at Berkeley, Berkeley, CA 94720, USA; 3 Department of Chemical Engineering, University of California at Berkeley, Berkeley, CA 94720, USA; 4 Physical Biosciences Division, Lawrence Berkeley National Laboratory, 1 Cyclotron Rd, Berkeley, CA 94720, USA

## Abstract

**Background:**

The transition from viral latency to lytic growth involves complex interactions among host and viral factors, and the extent to which host physiology is buffered from the virus during induction of lysis is not known. A reasonable hypothesis is that the virus should be evolutionarily selected to ensure host health throughout induction to minimize its chance of reproductive failure. To address this question, we collected transcriptional profiles of *Escherichia coli *and bacteriophage lambda throughout lysogenic induction by UV light.

**Results:**

We observed a temporally coordinated program of phage gene expression, with distinct early, middle and late transcriptional classes. Our study confirmed known host-phage interactions of induction of the heat shock regulon, escape replication, and suppression of genes involved in cell division and initiation of replication. We identified 728 *E. coli *genes responsive to prophage induction, which included pleiotropic stress response pathways, the Arc and Cpx regulons, and global regulators *crp *and *lrp*. Several hundred genes involved in central metabolism, energy metabolism, translation and transport were down-regulated late in induction. Though statistically significant, most of the changes in these genes were mild, with only 140 genes showing greater than two-fold change.

**Conclusion:**

Overall, we observe that prophage induction has a surprisingly low impact on host physiology. This study provides the first global dynamic picture of how host processes respond to lambda phage induction.

## Background

Bacteriophage lambda has been studied for over 50 years and has served as a model for understanding genetic networks, control and development. Lambda is a temperate phage capable of undergoing divergent developmental pathways: lysis and lysogeny. Lytic development is lethal to host *Escherichia coli*, resulting in amplification and release of progeny phage. In the lysogenic state the phage integrates into the host chromosome, where it can silence lytic promoters and replicate quiescently as a prophage. Induction of lysis from the lysogenic state can be triggered by agents that damage DNA or interfere with replication, such as mitomycin C and UV light. The gene regulatory network underlying the lambda lifecycle has been studied in exhaustive detail, yet the switch continues to reveal new levels of complex regulation [1]. The mechanistic details of this switch have been elegantly reviewed elsewhere [2].

The lambda gene regulatory network is composed of both phage and host factors, many of which interact with each other [3]. Host proteases, replication, transcription and translation machinery are necessary for phage replication and development. Proteins that interact with lambda antiterminator N play an essential role in regulating the temporal expression of delayed and late phage genes. Several host factors have been shown to alter phage gene expression by unknown mechanisms. For example integration host factor (IHF) appears to enhance transcription at lambda promoter P_L _[4]. Lambda gene products can, in turn, alter the state of host gene expression. For example, the lambda repressor CI directly represses *pckA*, a host gene involved in glucogenesis [5]. Additionally, lambda-N can antiterminate host transcripts in the galactose operon region during prophage induction, resulting in elevated levels of galactose enzymes [6]. Non-essential phage gene products of unknown function appear to alter host physiology, cell-cycle and macromolecular synthesis [7-9]. Overall, lambda development is sensitive to the physiological state of the host and possibly to stochastic fluctuations in host and phage protein levels [9-11].

The focus of this study is to identify interactions between host and phage during a dynamic phage process. These interactions are also of interest to the emerging field of synthetic biology in which new genetic circuits are engineered into cells, often from parts found in lambda [12-14], and must be designed to have minimal impact on the host. In the lysogenic state lambda expresses a small set of proteins that, among other things, repress lysis and confer fitness to the host while perturbing little else in host function [15]. This makes sense since advancing host fitness favors survival of the prophage. On the other hand, during lytic growth there is no such evolutionary concern for the host, other than a selection pressure to maintain host infrastructure for producing progeny phage. Hence, we wondered how host functions are modulated by the lambda phage gene expression program.

To address this question, here we characterize the effect of prophage induction on host physiology. Since much of the lambda regulatory circuitry is controlled at the transcriptional level, we surveyed gene expression profiles of the *Escherichia coli *and lambda phage transcriptomes throughout the timecourse of prophage induction by UV light. We constructed a whole-genome cDNA *E. coli*/bacteriophage lambda microarray, representing 99% of 4290 *E. coli *open reading frames and 66 predicted lambda open reading frames. We characterized the response of wild-type lambda lysogens and non-lysogens to UV light, and studied the impact of prophage induction on phage and host gene expression.

## Results

### Expression profiles of *Escherichia coli* and bacteriophage lambda ORFs

To evaluate gene expression of host and phage in a lysogen and during prophage induction, total RNA was harvested at 20-minute intervals from log-phase cultures of wild-type lysogenic and non-lysogenic *E. coli*. RNA samples and genomic DNA were fluorescently labeled and competitively hybridized on cDNA arrays containing 99% of *E. coli *open reading frames (ORFs) and 66 predicted ORFs of bacteriophage lambda. Lysogenic and non-lysogenic wild-type *E. coli *were compared at each time point, via a genomic control. Indirect comparison to a genomic DNA reference was useful for making multiple comparisons within and between the two strains.

**Figure 1:**
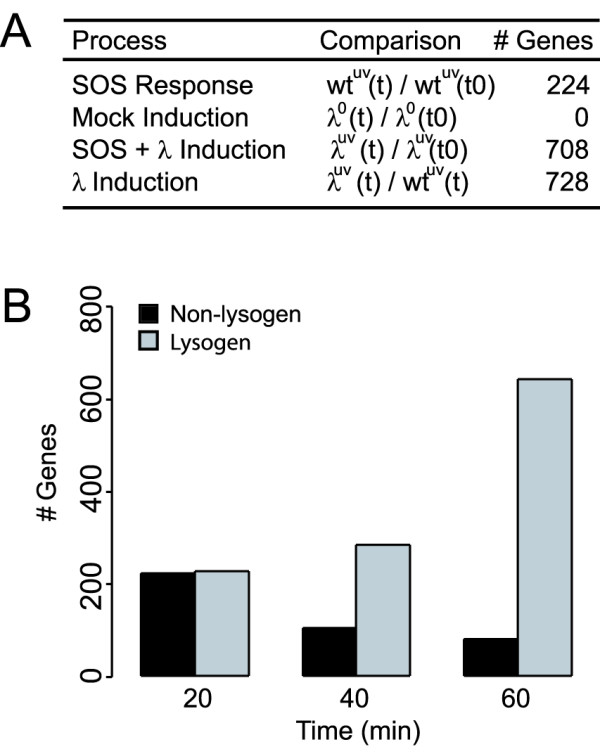
(A) Table summarizing altered gene expression in each timecourse. The total number of changing genes across all times is listed. 1. Wt^UV^(t)/wt^UV^(t0) refers to the SOS response of wild type *E. coli *following UV irradiation, relative to log-phase growth, 2. Lambda^0^(t)/lambda^0^(t0) refers to the mock induction protocol, relative to log-phase growth, 2. Lambda^UV^(t)/lambda^UV^(t0) refers to lambda lysogens following UV irradiation, relative to log-phase growth, 4. Lambda^UV^(t)/lambda^UV^(t) ratios are lambda lysogens relative to wild type non-lysogens. (B) Number of significantly changing host genes in lysogenic and non-lysogenic *E. coli*. Black = non-lysogenic *E. coli *response to UV light, Grey = lysogen-containing *E. coli *response to UV light.

We evaluated three time courses to distinguish the responses of host and phage to the following conditions: irradiation of non-lysogens with UV light, irradiation of lysogens with UV light, and a "mock induction" of lysogens in which cells were taken through the induction protocol but were not irradiated. *E. coli *genes were regarded as having statistically significant differential expression if they met the following criteria: average fold change > 1.4 and p-value ≤ 0.05 (see Methods). By applying these criteria to each time course we identified differentially changing genes for each condition (Figure 1).

### Gene expression of *Escherichia coli* and bacteriophage lambda in the lysogenic state

We observed eighteen *E. coli *genes to have significantly altered expression levels in lambda lysogens relative to uninfected bacteria (Table 1). Many of these changes were consistent with other genes in the same operon. Eleven of these transcripts encoded transport-related gene products, including the proline ABC transporter (*proWX*), the phosphate ABC transporter (*pstB*), spermidine transport permease (*potCB*), and the l-lactate utilization system (*lldPR*). Six transcripts were of unknown function. Only three significantly changing *E. coli *genes had expression differences greater than two-fold (*lldP, lldR *and *yccA*). It has previously been shown that *pckA *is repressed in lysogen-containing bacteria [5]. We do not observe lysogen-specific repression of *pckA *in our analysis because the gene is repressed in both wild type and lysogenic strains under our growth conditions, in which glucose is present.

**Table 1:** Significantly expressed genes in lambda lysogens, relative to non-lysogenic *E. coil *during log-phase growth. ^a ^Log_2 _ratio of lysogen relative to wild type *E. coli*. ^b ^Genes are significant if p-value ≤ 0.05, indicating 95% confidence.

**Gene**	**Log_2_Ratio^a^**	**Significance^b^**	**Product**
*yccA*	1.00	4.75 e-2	Unknown
*b4176*	0.96	7.70 e-3	Unknown
*proWX*	0.95	2.64 e-2	Proline ABC transport system
*purA*	0.80	4.48 e-2	Adenylosuccinate synthetase
*oppABC*	0.77	2.93 e-2	Periplasmic binding protein
*b1117*	0.68	4.17 e-2	Unknown
*potCB*	-0.62	9.95 e-3	Spermidine transport permease
*b1555*	-0.58	4.30 e-2	Unknown
*narY*	-0.60	4.93 e-2	Nitrate reductase 2, beta subunit
*pstB*	-0.61	3.08 e-2	Phosphate ABC transporter
*yafOP*	-0.73	2.74 e-2	Unknown
*lldPR*	-1.45	2.30 e-2	L-lactate utilization
*Lambda-CI*	4.14	4.21 e-4	Lambda repressor CI
*Lambda-RexAB*	3.22	3.88 e-6	Phage exclusion
*Lambda-G*	1.31	3.77 e-2	Tail component
*Lambda-orf63*	0.97	4.04 e-2	Unknown
*Lambda-bet*	0.80	4.85 e-2	Recombination
*Lambda-xis*	0.62	2.80 e-2	Excision
*Lambda-T*	0.54	2.63 e-2	Tail fiber protein

In the lysogenic state most of the lambda genome is silenced by the repressor CI. At 95% confidence, we detected significant expression of eight phage genes (Table 1). The most highly expressed genes were on the P_RM _operon: *cI*, *rexA *and *rexB*. Phage genes *lom *and *bor*, virulence factors known to be expressed in a lysogen [15], were not detected at significant levels in our microarrays. *Bor *is not detected because the coding region in our strain was deleted and replaced with an antibiotic marker. It is unknown why *lom *is not expressed at detectable levels in our experiments: little is known about regulation of the *lom *promoter; it may be repressed under the growth conditions in this study. A previous study by Chen *et al*, examining gene expression of the lysogenic state during growth on minimal media, observed high expression levels of *kil*, *int*, and *gpE*, in addition to *cI*, *rexAB*, *lom *and *bor *[5]. Our expression study confirms the Chen *et al *data for *cI *and *rexAB *gene expression but differs in two ways: first, we do not observe significant expression of *kil*, *int *or *gpE*; second, we observe low-level induction of *gpG*, *gpT*, *bet*, *xis*, and *orf63*. Despite these differences, changes in non-lysogenic genes in both studies were small (less than two-fold) and the measurements are particularly sensitive to noise, as expression ratios compare samples with and without the lambda genome.

### Gene expression of bacteriophage lambda during prophage induction

Prophage induction is known to proceed in a temporal cascade of regulation, initiated by depletion of repressor CI at upstream binding sites in O_R _and O_L_. Upon exposure to UV light, CI undergoes an auto-cleavage reaction stimulated by an activated form of RecA, probably activated by binding to single-stranded DNA at sites of damage [16]. Degradation of CI results in the de-repression of early lytic promoters P_R _and P_L _and transcription of anti-terminators N and Q. N is a regulator of delayed-early transcripts that anti-terminates sites in both P_R _and P_L _operons, allowing transcription of the full program of delayed early genes including replication and excision machinery. The Q gene product anti-terminates the late P_R_' lytic promoter. Accumulation of Q is necessary for transcription of capsid, assembly, tail and lysis genes from P_R_'.

**Figure 2:**
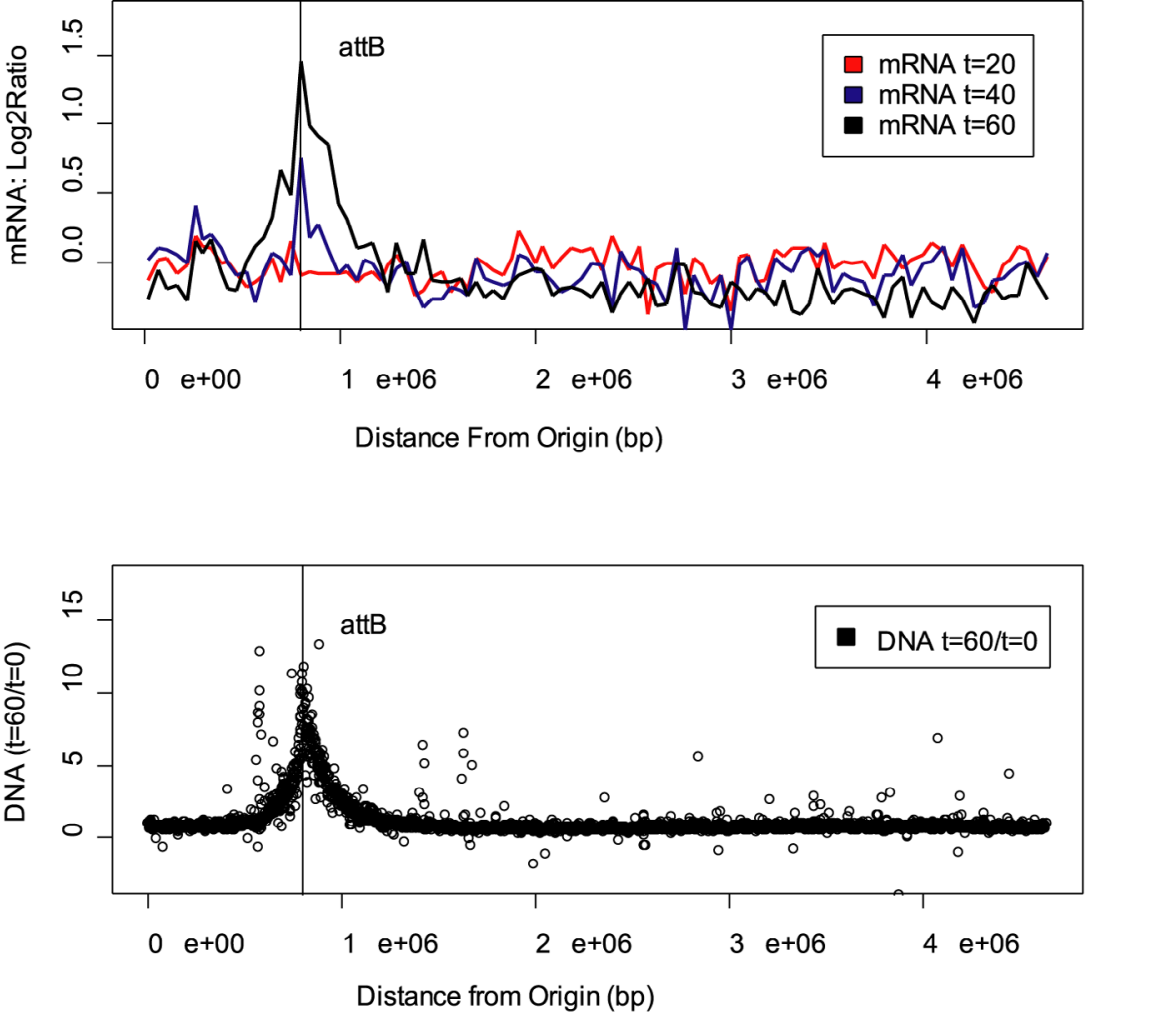
**A) **Diagram of the linear (integrated) lambda phage genome, color-coded by lifecycle stage (blue = lysogenic, yellow = early lytic, red = late lytic). **B) **(wild type phage) and **C) **(Lambda-P27): gene expression ratios during prophage induction are shown relative to an untreated "mock induction" control and log_2 _transformed. Genes arranged by order on the lambda genome.

To characterize the lambda gene expression program during induction, transcript levels at each time point after induction were compared to basal expression levels of cells undergoing a mock induction protocol. Basal expression levels are detectable above background noise in all lambda probes due to a low frequency of spontaneous de-repression. Most measured phage transcripts were significantly up-regulated during the time course of prophage induction (Additional File 1). Early in induction, very little phage gene expression was detected. At 20 minutes several delayed early and early lytic genes were detected at significant levels including *N*, *cro*, *cIII*, *gam *and *bet*. At 40 minutes all early lytic and some late lytic genes were significantly upregulated. By 60 minutes, all late phage genes were highly expressed (Figure 2A and 2B). At this time in induction, 20% of the total detected transcripts encoded phage proteins (Additional File 5).

We also grouped the phage genes by average linkage hierarchical clustering (Additional File 6). In the expression data presented here, four main clusters of genes were clearly distinguished. Each cluster corresponded to different developmental stages of the phage lifecycle: lysogeny, early lytic, and two clusters of lytic transcription. The observed expression timing of phage genes proceeded in a temporal cascade, as expected.

### Verification of lambda phage gene expression by RT-PCR

Several genes from the lambda phage genome were analyzed by RT-PCR, including repressors *cI *and *cro*, *P*, *N*, *Q*, and *lom*. One of these genes, *P*, was not present on our arrays but exhibited the same qualitative behavior as other early lytic genes. RT-PCR data (Additional File 2) indicate that the fold-change of several genes was higher than that measured on microarrays, but that the direction of change was the same.

### *E. coli* gene expression is insensitive to "mock irradiation" protocol

As a negative control, to confirm that the irradiation protocol has no effect on cell physiology, irrespective of UV light, we performed "mock inductions" on lysogens using a standard protocol for inducing phage. As expected, none of the host or phage transcripts were differentially expressed over the course of 60 minutes following the mock induction protocol.

### Gene expression program of the *E. coli* SOS response

The *E. coli *SOS response has been studied extensively and global expression levels have been profiled using microarrays [17,18]. In the transcriptional profiling study by Courcelle *et al*, a dose of 40 J/m^2 ^was applied to induce an SOS response in wild-type *E. coli*. In results presented here, we selected a dose of 10 J/m^2 ^UV light because it elicits a less intense SOS response but is a sufficiently high dose to induce most lysogens (see Methods). We observed differential regulation of 224 genes in wild-type *E. coli* following irradiation by UV light (Additional File 3). In our study, 21 genes known to be regulated by LexA were significantly up-regulated 20 minutes after UV exposure, in agreement with prior studies [17,18]. The gene expression profiles of the UV-response genes in lysogenic and non-lysogenic *E. coli *were similar for the first 20 minutes; at 40 and 60 minutes the response diverged, presumably due to the impact of prophage induction (Figure 1B). Our results are not directly comparable to other studies as we used different strains and media and a much lower UV dose.

### Host gene expression during prophage induction

To evaluate significantly changing host genes during prophage induction, we compared temporal expression profiles of lysogenic and non-lysogenic *E. coli *at 20 minute intervals, following exposure to UV light. Using the selection criteria described previously, we observed differential expression of 728 host genes (Additional File 4). Most changes were small (less than 2 log_2_-fold) and occurred late in induction, with reduced expression of genes in several COG functional groups.

### Transcriptional enhancement observed near lambda integration site during prophage induction

**Figure 3:**
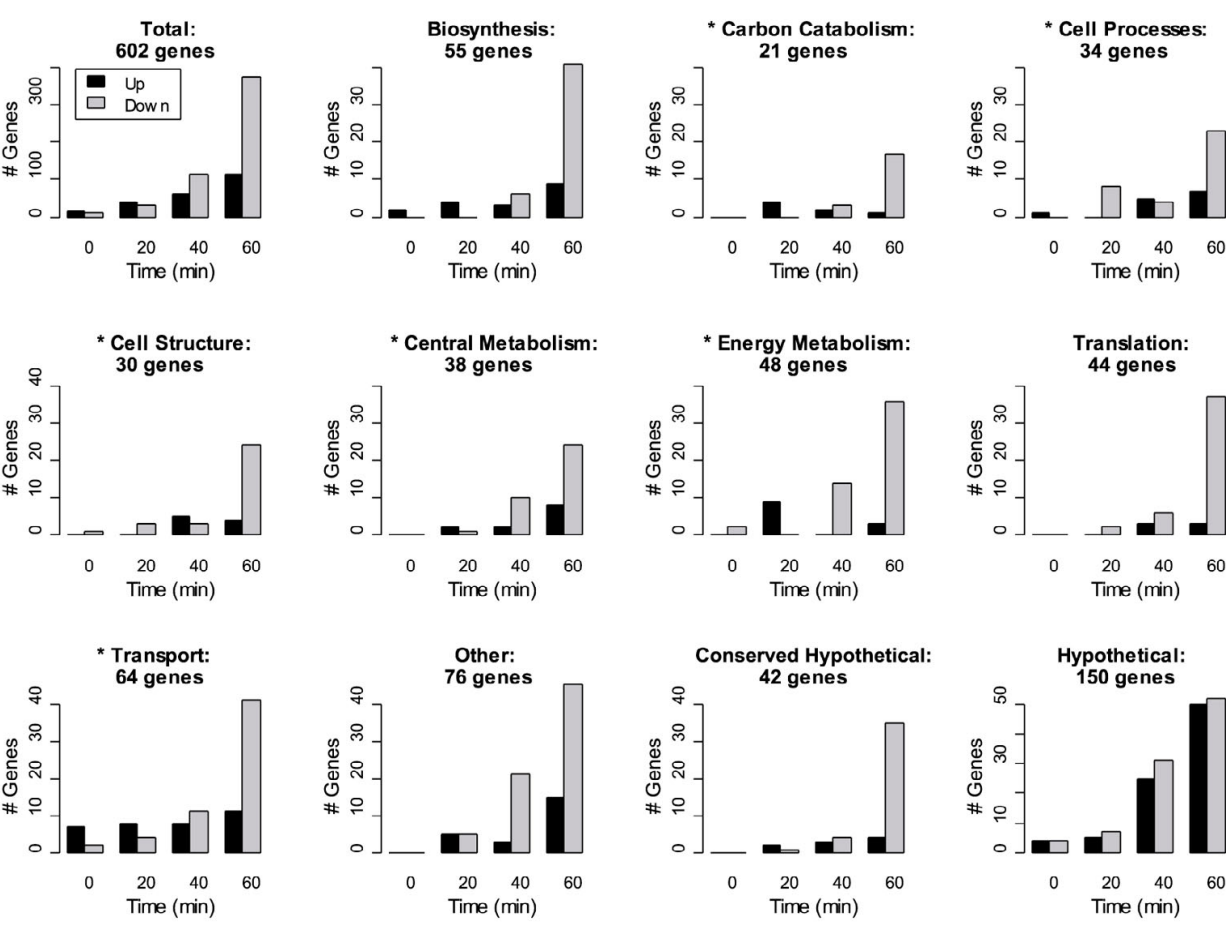
*Escherichia coli *lambda lysogen DNA and average transcript levels after treatment with 10 J/m2 UV light. The x-axis is the position of genes on the *E. coli *chromosome. The *E. coli *origin is at the 0 position on the x-axis. The lambda integration site *attB *is indicated by the vertical line. The y-axis is the log ratio of treated to untreated cells. **A)**. Average transcription (100 bins) along the *E. coli *chromosome at 20, 40, 60 minutes after exposure to UV light. **B**). Ratio of DNA 60 minutes after treatment with UV light relative to DNA of untreated cells.

Genes in the *gal *operon, adjacent to the phage integration site *attB*, are amplified during prophage induction in *E. coli*; this amplification has been attributed to increase in gene dosage due to escape replication from the prophage origin [19] and trans-acting antitermination by lambda-N [6,20]. In data presented here, transcription of *gal *operon genes was enhanced nearly 13-fold in lysogens relative to non-lysogens, as expected. In addition to the *gal *operon, we observed enhancement of transcription in a 300-kb region surrounding the prophage integration site, *attB *(Figure 3A). The boundaries of this region were defined by binning the average expression fold-change at defined intervals. In the *attB *region, 126 genes demonstrated 1.4-fold or higher up-regulation; no genes in the region were down-regulated. As distance from *attB *increased, the average fold-change of host genes decreased.

### Host DNA adjacent to attB is amplified at high copy number

It was recently demonstrated that induction of lambdoid phages in *Salmonella enterica *can lead to amplification of large regions of the host genome next to phage integration sites via escape replication [21]. Sternberg observed escape replication in bacteriophage lambda extending to chromosomal markers up to 10 min from the *attB *integration site [22]. To examine the impact of prophage induction on DNA copy number under our conditions, we competitively hybridized genomic DNA from untreated lysogens with DNA isolated from lysogens one hour after irradiation with UV light. Over 400 genes in a 400-kB region surrounding *attB *showed an increase in copy number (Figure 3B), indicating that replication is initiating at the lambda origin and extending into the host chromosome. As distance from the prophage increases the DNA copy number is reduced. Increased gene dosage via escape replication from *attB *likely contributes to enhanced levels of host transcripts in this region.

### A replication-defective phage has reduced impact on the host in the attB region

To evaluate the impact of DNA copy number on transcriptional response in the *attB *region, we examined the gene expression profile of a UV-induced lambda prophage defective in replication. In comparison to non-lysogenic bacteria, 350 genes were differentially regulated during the course of prophage induction, less than half that observed in wild type phage. In the *attB *region we observed differential regulation of 21 genes, with no evident bias for upregulated genes or position (Table 2). We did not observe an increase in DNA copy number in this region (data not shown). Most genes with known function encoded protiens involved in energy metabolism. The direction of change for these genes was negative, consistent with our observation of energy metabolism genes during induction of wild type phage discussed below.

**Table 2:** Differentially expressed host genes in the *attB *region in lambda-P27 replication-defective mutants.

**COG**	**Gene**	**Lambda-WT**	**Lambda-P27**
Cell division	*ftsK*	+	+
Energy metabolism	*gltA*	+	-
	*sdhCDAB*	+	-
	*sucBCD*	+	-
Transport	*modB*	+	+
Transposons	*b0712–0713*	+	-
	*b0725*	+	+
	*b0762*	+	+
	*ybhD*	+	+
	*b0770–b0771*	+	+
	*b0807*	-	+
	*b0830–0831*	-	+
	*b0845*	-	+

### Functional categorization of differentially regulated host genes during prophage induction

**Figure 4:**
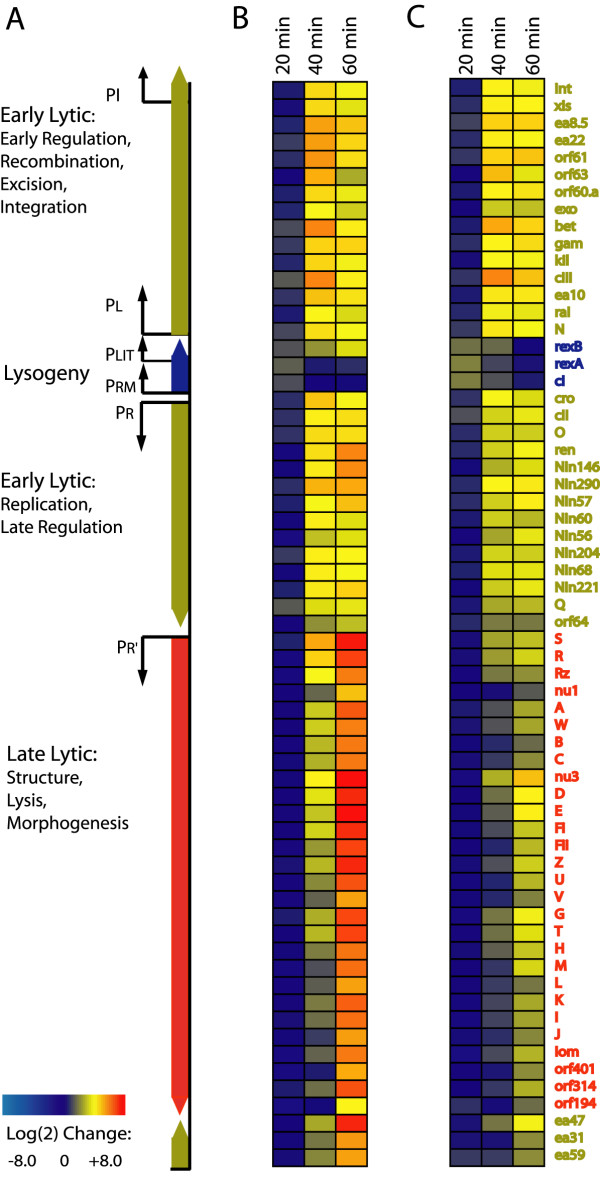
Functional categorization of *E. coli *genes during lambda phage induction. Histograms count number of genes significantly up-regulated (black) or down-regulated (grey) at each time interval. Genes were grouped according to the NCBI COG classification scheme[49]. Categories with an (*) were enriched in down-regulated genes (Fisher exact test, false discovery rate < 0.05): carbon catabolism, cell processes, cell structure, central metabolism energy metabolism, and transport.

Excluding the up-regulated genes in the *attB *region, we examined the functional categorization of the remaining 602 genes responsive to lambda phage. 150 of these genes were putative open reading frames with no known function. Few genes exhibited altered expression at early timepoints; the most significant change 20 minutes after UV exposure was 8-fold induction of the *fruABK *operon, involved in fructose transport and metabolism. Most host genes were differentially regulated at later timepoints (Figure 4). A Fisher exact test (false discovery rate ≤ 0.05) demonstrated that after sixty minutes six out of 23 functional groups were enriched in down-regulated genes: biosynthesis, carbon catabolism, cell processes, cell structure, energy metabolism and transport. No functional groups were enriched in up-regulated genes.

In addition to this functional coherence, several genes known to interact with lambda showed altered transcriptional regulation. It was previously shown that production of early lambda gene products leads to increased levels of heat shock gene products [23]. Induction of the heat shock regulon is mediated indirectly by lambda-CIII, which inhibits protease FtsH and thereby stabilizes σ32 (*rpoH*) and other FtsH-sensitive proteins [24,25]. In data presented here, we observed altered transcription of several genes in the heat shock regulon, including molecular chaperones encoding proteins essential for bacteriophage replication and morphogenesis [26,27]. Transcription of *dnaK*, *mopA *(*groEL*), *mopB *(*groES*), *htpGX *and *hslUV *was significantly enhanced forty minutes after induction and repressed at sixty minutes. Expression of additional heat shock genes *htrA *and *hsiT *was increased four-fold at 40 and 60 minutes after induction. Up-regulation of the heat shock regulon coincided with peak levels of the *lambda-cIII *transcript.

Expression of accessory genes on the lambda-P_L _operon has been shown to alter the *E. coli *cell cycle by transiently blocking initiation of replication and cell division [7,9]. We investigated the extent of this effect on host gene expression during lambda induction and found altered expression of several genes associated with initiation of replication and cell division. Cell division genes *ftsJ, sulA*, *tig*, *dicB, minD *and *xerC *were down-regulated late in induction. The *tig *gene encodes Trigger Factor (TF), an important molecular chaperone involved in cell division [28,29]. Changes in TF levels cause defective cell division [28]. XerC is a recombinase that effects chromosome segregation at cell division. Inhibitors of cell division, d*icB*, *sulA*, and *minD*, were down-regulated at forty and sixty minutes. Increased expression was observed at sixty minutes in genes involved in cell division (*ftsK*) and initiation of replication (*mukB *and *seqA*). These genes are located near the lambda integration site, so any change in their expression levels late in induction is complicated by the increase in copy-number in this region.

### Differential regulation of two-component signaling pathways and global regulators

We observed altered regulation of several genes in the Cpx and Arc two-component regulatory systems. The Cpx response network is estimated to regulate the expression of over 100 genes, involved in functionally diverse processes such as management of protein distress, motility and chemotaxis, biofilm formation and response to envelope stress [30]. *cpxA *and *cpxR*, co-transcribed on an operon activated by σS (RpoS) and CpxR-P [31,32], were upregulated in lambda lysogens 40 minutes after induction. In addition to *cpxAR*, 23 genes in the Cpx regulon were differentially regulated in our data. These genes encode proteins involved in diverse physiological activities, including envelope distress (*ompCF*), management of protein distress (*dnaK*, *ftsJ*, *hlpA*, *hslTS*, and *tig*), the starvation response (*sspA*), the PTS system (*manXY*, *ptsI*), glycine cleavage (*gcvTHP*), and regulation of sigma-E (*rpoErseAB*). Of the 7 genes known to be under positive control of Cpx-P, six were upregulated including *cpxAR*, *yihE-dsbA*, and *rpoErseAB*. The direction of Cpx regulation is unknown for the remaining genes. There is overlap between genes expressed in the Cpx and RpoH regulons, and it is speculated that these systems, along with the RpoE regulon, respond to a variety of stresses in a concerted manner [30].

The Arc system responds to respiratory growth conditions to modulate expression of an estimated 100–150 genes, including metabolic regulons [33,34]. Normally activated under anaerobic conditions, the Arc response can be also be activated under aerobic conditions in the presence of reducing agents [35]. We identified differential regulation of 46 genes in the Arc regulon. Nearly all the genes were repressed 60 minutes after prophage induction, and all but five were expressed in the same direction of change consistent with previously published data. Several multi-gene operons known to be repressed by phosphorylated ArcA were down-regulated including *aceAB*, *agaCDI, cyoABCDE*, *lctPRD*, *nuoABCEFGHIJKLM*, and *treBC*. *surA*, encoding a protein that mediates stress-induced survival, was also downregulated. Further experimentation is required to test whether these shifts in gene expression are a response mediated by the Arc system or a pleiotropic stress response.

In addition to altered transcription of the Cpx and Arc regulons, we observed changes in other global regulators in lambda lysogens, including global regulators *crp *and *lrp*. Lrp was upregulated at 40 and 60 minutes. The *crp *transcript was downregulated at 40 and 60 minutes. Since these changes occurred late in induction, little effect on downstream pathways was observed before bacteria started lysing *en masse*.

## Discussion

In this study we measured gene expression changes in wild-type *Escherichia coli *with and without an integrated prophage, during exponential growth and following exposure to a mild dose of UV light. We observed that the lambda phage genes were up-regulated in a temporal cascade. We also found that 728 *E. coli *genes were differentially expressed in response to prophage induction. Most of these changes, while significant, were small in magnitude and occurred late in induction.

It was expected that the phage gene expression would progress in a temporally organized manner and for the most part, lambda behaved as expected. We observed lysogenic transcripts to be up-regulated 20 minutes after induction. This result was confirmed by RT-PCR. In models of promoter activity at P_RM _by Dodd *et al*, a decrease in repressor concentration from lysogenic levels can stimulate activity of P_RM _[36]. The increase in lysogenic transcript abundance could result from transient de-repression of the P_RM _promoter or by an increase in gene dosage due to lambda DNA replication.

The host response to lambda phage induction by UV light is more complex than previously thought, causing shifts in expression of 728 genes. That complexity was compounded by the necessity to consider multiple conditions as controls. In a mock induction control, we found that our induction protocol does not have a significant impact on host or phage gene expression. Controlling for response to UV light exposure, we found the SOS response in non-lysogenic and lysogenic *E. coli *to be in good agreement with previous findings.

Of the genes that were up-regulated during prophage induction, nearly all were proximal to the *attB *integration site, likely due to an increase in copy number of the DNA in that region. Escape replication appears to be a common strategy for some, but not all, lambdoid phages [21,37]. Prophage induction with a replication-defective mutant prophage eliminated both transcriptional and DNA copy-number enhancement in this region.

Several known mechanisms of host-phage interaction were confirmed by our data, including induction of the heat shock regulon, escape replication and transient inhibition of cell division and initiation of replication. It has been speculated that these interactions benefit lytic development by increasing phage gene dosage and the available pool of host resources.

At the peak of late lytic-phase lambda gene expression, several hundred host genes were down-regulated. These genes were involved in diverse cellular processes including biosynthesis, cell structure, central and energy metabolism, and transport. Due to replication and high levels of phage gene expression, one would expect that expression of many host genes would be altered simply by mass action, especially late in induction when approximately 20% of the transcriptional output encodes phage proteins. A follow-up experiment in which late lambda gene expression is blocked may reveal other interesting and specific effects of early lambda gene products.

In results presented here, late lambda phage induction appears to induce general stress response pathways in the host, including the Arc and Cpx two-component systems, and global regulators *crp *and *lrp*. The Arc system is activated by alterations in the redox state of the cell. The Cpx regulon is a pleiotropic stress response system, responsive to diverse kinds of stress including envelope and nutritional distress. The Cpx system interacts with the heat shock regulon, also upregulated during lambda induction. *Crp *and *lrp *are both sensitive to the nutritional state of the bacterium. Depletion of cellular resources by the phage, along with envelope distress due to pending cell lysis, may contribute indirectly to these changes in host gene expression. Global regulators can be induced by various intra- and extracellular cues, so it is unclear whether expression of these genes is pleiotropic or mediated by a specific factor. The host does not appear to deploy specific defense mechanisms in response to prophage induction at the gene expression level; rather the observed transcriptional shifts are directed by the phage, both directly and indirectly.

## Conclusion

This study identified 728 *E. coli *genes responsive to lambda phage induction by UV light and confirmed known host-phage interactions. Most host genes are differentially expressed late in induction at low fold-change. Overall, prophage induction had a surprisingly low impact on host gene expression. These results imply that bacteriophage lambda, as a module, is relatively independent from its host during prophage induction, exerting relatively little load on host physiology until the lytic process is nearly complete.

## Methods

### Media, bacterial strains, and growth

Cultures were grown in LBGM: LB supplemented with 1 mM MgSO4, 0.2% glucose, and appropriate antibiotics. TMG was 10 mM Tris-HCl, pH 8.0, 10 mM MgSO4, 10 ug/ml gelatin. *E. coli *strains MG1655, JL2497 and JL5932 were used for microarray studies. MG1655 DNA was spotted on the cDNA microarrays. JL2497 is a wild-type strain, derivative of N99; N99 is the same as W3102, which was derived from W3110 [38,39]. JL5932 is JL2497 containing a wild-type lambda lysogen [40]. JL2497-Lambda-P27 is JL2497 containing a mutagenized lambda lysogen defective in replication. Lambda-P27 has an amber mutation in the 27^th ^codon of the P gene, and was mutated

### Recombineering

Lambda-P27 is wild type lambda phage defective in replication. We generated a suppressor-sensitive mutation in lambda-P via i*n vivo *recombineering with an 70-mer oligo [41]. The oligo was designed to engineer a point mutation in the 27^th ^codon of the P gene, introducing a UAG termination codon into the sequence. Sequence = CTGCGCTACCTGCTGTACCTGCGGCTTTTCGTCCTACTGTTCCGGCATGTTGTTGGCGATCCGACGCATC. The amber mutant was identified by screening for growth on wild type and amber suppressor strains. JL2497 containing lambda-P27 lysogens were selected for by growth on plates with appropriate antibiotics.

### Prophage induction experiments

Dose-response curves were generated as previously described [40], and we measured the set point for wild type lysogens to be 5 J/m^2 ^(data not shown). Cells were grown in LBGM to 2 × 10^8/ml (OD_600 _= 0.4), chilled, centrifuged, resuspended in TMG at 2 × 10^8/ml, and irradiated at 254 nm in dim ambient light at a dose of 10 J/m^2 ^at 0.2 J/m^2^/s. 10 J/m^2 ^is a mild dose at which phage induce at a high frequency (around 80%). Cells were centrifuged, resuspended in 37°C LBGM at 2 × 10^8/ml, and shaken for 80 min at 37°C in the dark. Aliquots were taken for OD measurements and RNA isolation at 20 minute intervals. Since lambda phage gene expression during induction is smooth and monotonic increasing, a 20-minute sampling interval was sufficient to capture the dynamics. The time points were chosen between 0 and 60 minutes, to reflect the observed timescale of prophage induction in the literature and to avoid sampling after cell death. In our experiments cells began to lyse between 60 and 70 minutes after irradiation (data not shown). At least three biological replicates of each time course were repeated.

### cDNA microarrays

cDNA microarrays were manufactured in-house on Telechem SuperAmine substrates using a GeneMachines OmniGrid. Printing protocols are described in MGuide [42]. Arrays contained 4250 open reading frames (ORFs) representing 99% of *E. coli *ORFs according to Blattner [43] and 66 lambda ORFs. Sequences of the lambda primer set are available from the corresponding author aparkin@lbl.gov. Each microarray contained *E. coli *ORFs spotted in duplicate and lambda ORFs spotted in quadruplicate. mRNA levels were determined by two-color parallel hybridizations relative to labeled and reverse transcribed genomic DNA, isolated in stationary phase.

### RNA isolation and cDNA labeling

Cells were harvested at 20 minute intervals and pellets were immediately frozen in liquid nitrogen. Total RNA was isolated and purified using an RNeasy mini kit, and treated with on-column DNase (Qiagen). RNA samples were quantified using an Agilent Bioanalyzer, and stored at -80°C for later use. 30 ug RNA was reverse transcribed into cDNA using SuperScriptII (Invitrogen) using the manufacturer's protocol. Aminoallyl-labeled dUTP (Ambion) was included in the RT reaction mix in a 4:1 ratio of aa-dUTP:unlabeled dUTP. The labeling reaction was treated with 1 N NaOH for 10 minutes at 70C, then neutralized with 1 N HCl. cDNA was purified by eluting Microcon-30 columns (Millipore) three times with 500 ul sterile nanopure water. Dye coupling was achieved with Ambion's Alexa 555 and Alexa 647 dyes, using to the manufacturers protocol. cDNA concentration and labeling efficiency were measured with a Nanodrop. Equal amounts of labeled cDNA (Alexa 555) and labeled genomic DNA (Alexa 647) were resuspended in Ambion SlideHyb Buffer #3 and hybridized for 12 hours at 42°C on a Tecan HS4800 hybridization station.

### Genomic DNA labeling

Genomic DNA of JL5932 was prepared from fresh overnight cultures or from bacteria in logarithmic growth phase using Qiagen Genomic-tip 500/G kits (Qiagen). DNA was labeled with Alexa 555 or 647 according to the following protocol. 3 ug DNA was suspended in 18 ul water and 20 ul 2.5× random primer mix (Invitrogen BioPrime Kit). Mixture was boiled for 5 minutes, then chilled on ice. 8 μl aa-dNTP mix (1.25 uM dATP, dCTP, dGTP, 0.25 uM dTTP, 1 uM aa-dUTP) and 1 ul high concentrate Klenow fragment (Invitrogen BioPrime Kit) were added and reaction mixture was incubated for 3 hours at 42°C. Purification, dye coupling and subsequent treatment were identical to cDNA labeling protocols described above.

### Data analysis

Microarray images were processed with Axon GenePix 6.0 software. Raw and normalized log2-transformed data can be found online [44]. Data filtering and normalization were performed using the R statistical package. Background noise was subtracted from red and green channels, and each spot was assigned an R/G ratio. Data was normalized by scaling each ratio to the sum of all spots present on all replicates of each condition. Normalized data was log_2_-transformed and average signal intensities were calculated from at least four independent replicate experiments. *E. coli *genes with ≥90% nucleotide identity to λ phage genes were excluded from the analysis. Statistical analysis was performed using the OpWise open-source software for estimating noise based on behavior of genes in operons [45]. Genes displaying statistically significant differential expression met the following conditions: average fold-change ≥ 1.4 and OpWise p-value (p_OW_) indicated ≥ 95% confidence (p_OW _≥ .975 or p_OW _≤ 0.025). OpWise p-values are two-tailed; in this paper and in the supplementary material p-values are transformed for clarity: p = 1–2*|0.5-p_OW_|. Normalized data of lambda phage genes was clustered by average linkage hierarchical clustering within the Genesis microarray analysis software platform (Additional File 6) [46].

### RT-PCR

RT-PCR was used to confirm DNA microarray gene expression data for selected phage genes. Total RNA was extracted in independent cultures, exactly as described above. RNA quality and concentration were measured with an Agilent Bioanalyzer. One-step RT-PCR was performed with the SuperScript III Platinum SYBR Green One-Step qRT-PCR Kit, per the manufacturer's instructions. An annealing temperature of 55°C was used for a total of 45 cycles. Primers were designed using Primer3 software [47] and are available upon request from the corresponding author aparkin@lbl.gov.

## Supporting information

In adherence to MIAME reporting standards expression ratios, raw data and protocols are available online [44]. They will also be published in the NCBI GEO public data repository [48]. Detailed protocols are available upon request.

## Accession numbers

The GenBank  accession numbers for the genes and gene products discussed in this paper are included in the supporting information. The GenBank accession number for the *E. coli *MG1655 primer set is U00096.

## Supplementary Material

Additional File 1 Table S1: Viral gene expression during lambda phage induction. This table lists lambda phage gene expression changes during prophage induction by 10 J/m2 UV light. Transcript levels of UV-induced lambda lysogens are expressed relative to basal expression levels of lysogens undergoing a mock-induction protocol. A is the GenBank accession number, H is the gene product name. B, D, and F are the log2 ratio of lysogens at time 20, 40, and 60 minutes, relative to lysogenic levels. C, E, and G are p-values for each log ratio. Click here for file

Additional File 5 Figure S1. Ratio of phage RNA to total RNA during prophage induction. The data shows the ratio of phage to total (host + phage) DNA during prophage induction. Click here for file

Additional File 6 Figure S2. Hierarchial clustering diagram of lambda phage genes following exposure to UV light. The expression ratios are relative to an untreated control and log_2 _transformed. Bars indicate clusters (1–4) of co-regulated genes. Click here for file

Additional File 2 Table S2. RT PCR of selected lambda phage genes during induction. Copy number is listed as a fold-change, relative to the lysogenic level of each gene. Click here for file

Additional File 3 Table S3. Host genes induced by exposure to 10 J/m2 UV light. This table lists *E. coli* genes induced by exposure to 10 J/m2 UV light. Column A is the GenBank gene accession number, B is the Blattner b-number, I is the gene name. C, E and G are log2 ratio of wild type *E. coli *at time 20, 40, 60 minutes, relative to bacteria in log-phase growth (time 0). D, F, H are the p-values for each log ratio. Click here for file

Additional File 4 Table S4. Host genes regulated during lambda phage induction by UV light. This table lists *E. coli *genes regulated during lambda phage induction by UV light. Column A is the GenBank gene accession number, B is the Blattner b-number, K is the gene name. C, E, G and I are log2 ratio of lysogens at time 0, 20, 40, 60 minutes, relative to non-lysogens. D, F, H and J are the p-values for each log ratio. Click here for file
