# Correction: Global analysis of host response to induction of a latent bacteriophage

**DOI:** 10.1186/1471-2180-13-183

**Published:** 2013-08-06

**Authors:** Robin E Osterhout, Israel A Figueroa, Jay D Keasling, Adam P Arkin

**Affiliations:** 1 Department of Bioengineering and Howard Hughes Medical Institute, University of California at Berkeley, Berkeley, CA, 94720, USA

## Correction

After the publication of this work [1], we became aware that the legends for Figures 2, 3 and 4 were not in the correct order.

The legends should be as follows:

**Figure 2:***Escherichia coli* lambda lysogen DNA and average transcript levels after treatment with 10 J/m2 UV light. The x-axis is the position of genes on the *E. coli* chromosome. The *E. coli* origin is at the 0 position on the x-axis. The lambda integration site *attB* is indicated by the vertical line. The y-axis is the log ratio of treated to untreated cells. **A)**. Average transcription (100 bins) along the *E. coli* chromosome at 20, 40, 60 minutes after exposure to UV light. **B**). Ratio of DNA 60 minutes after treatment with UV light relative to DNA of untreated cells.

**Figure 3:** Functional categorization of *E. coli* genes during lambda phage induction. Histograms count number of genes significantly up-regulated (black) or down-regulated (grey) at each time interval. Genes were grouped according to the NCBI COG classification scheme [49]. Categories with an (*) were enriched in down-regulated genes (Fisher exact test, false discovery rate < 0.05): carbon catabolism, cell processes, cell structure, central metabolism energy metabolism, and transport.

**Figure 4: A)** Diagram of the linear (integrated) lambda phage genome, color-coded by lifecycle stage (blue = lysogenic, yellow = early lytic, red = late lytic). **B)** (wild type phage) and **C)** (Lambda-P27): gene expression ratios during prophage induction are shown relative to an untreated "mock induction" control and log_2_ transformed. Genes arranged by order on the lambda genome.

## References

[B1] Osterhout RE Figueroa IA Keasling JD Arkin AP Global analysis of host response to induction of a latent bacteriophage BMC Microbiol 2007 7 82 10.1186/1471-2180-7-82 17764558 PMC2147009

