# Supplementary figures and images for: Global analysis of host response to induction of a latent bacteriophage
Source: BMC Microbiol. 2007 Aug 31;7:82. doi: 10.1186/1471-2180-7-82 (PMC2147009; doi:10.1186/1471-2180-7-82)

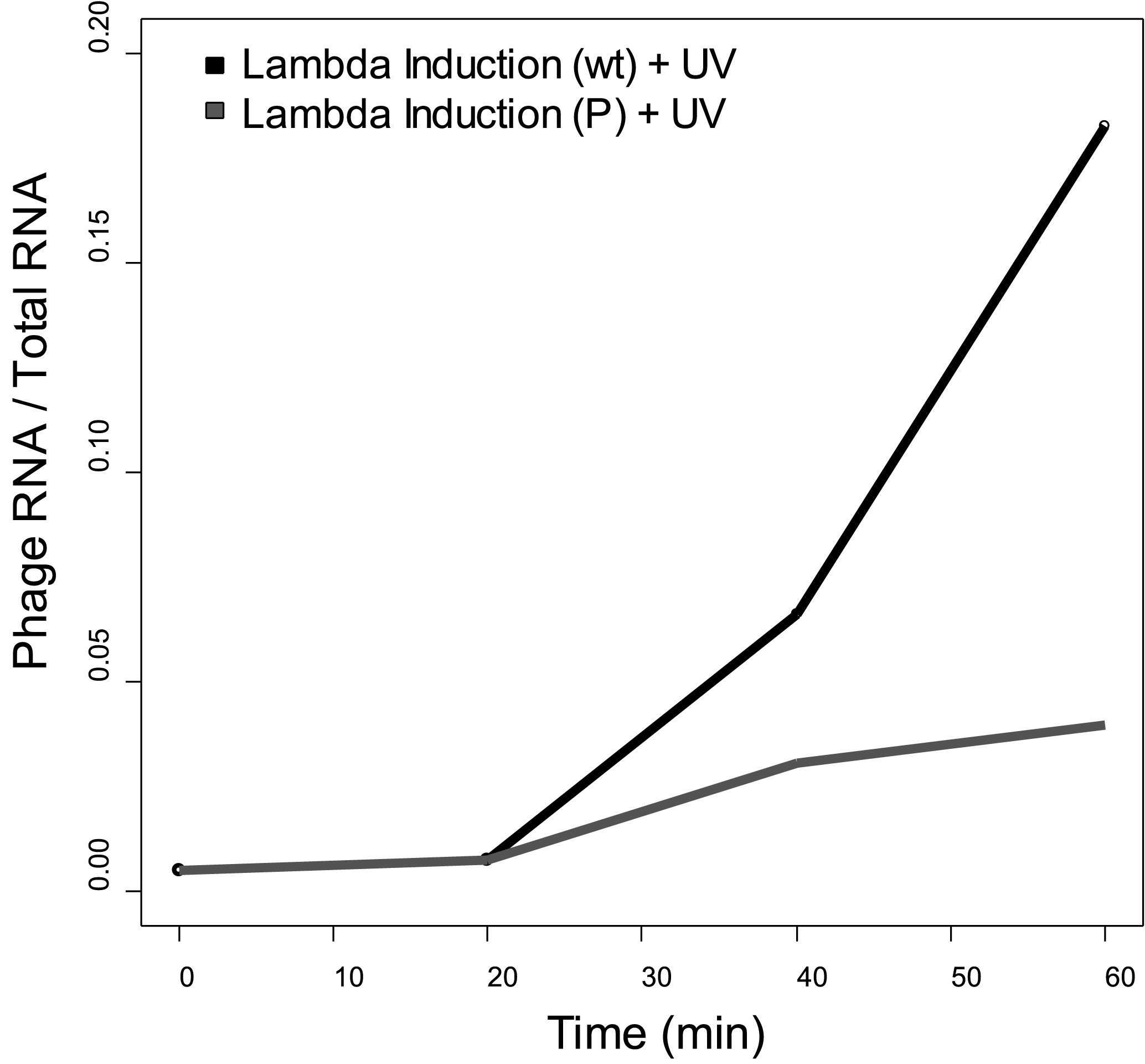

Supplement: Additional File 5 — Figure S1. Ratio of phage RNA to total RNA during prophage induction. The data shows the ratio of phage to total (host + phage) DNA during prophage induction. [file 1471-2180-7-82-S5.tiff]

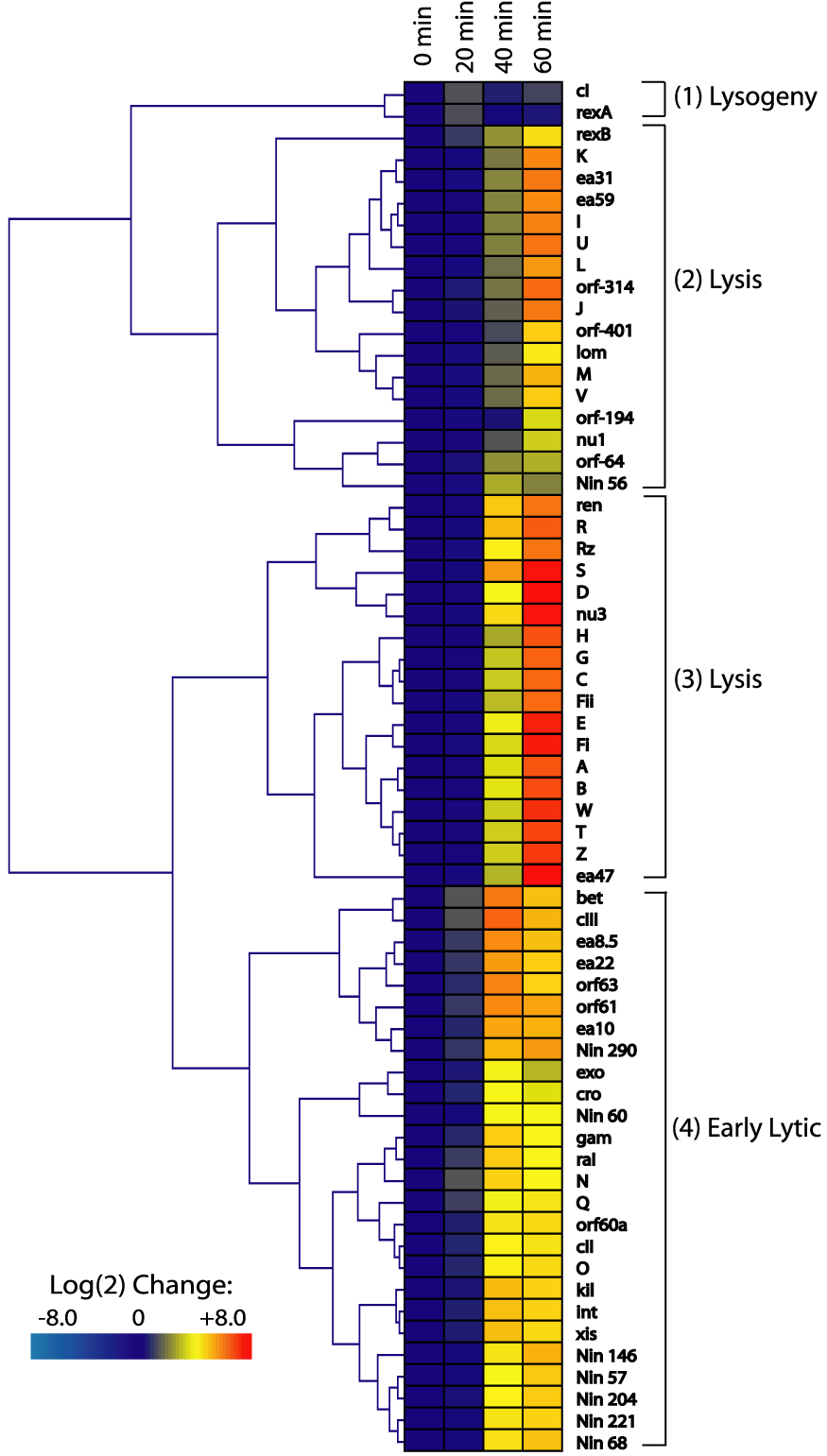

Supplement: Additional File 6 — Figure S2. Hierarchial clustering diagram of lambda phage genes following exposure to UV light. The expression ratios are relative to an untreated control and log2 transformed. Bars indicate clusters (1–4) of co-regulated genes. [file 1471-2180-7-82-S6.tiff]
